# Growth and Decay of a Planktonic Microbial Culture

**DOI:** 10.1155/2020/4186468

**Published:** 2020-01-24

**Authors:** Alberto Schiraldi

**Affiliations:** Department of Chemistry, Division of Physical Chemistry, University of Milan, Via Golgi 19, 20133 Milano, Italy

## Abstract

The paper shows that the phenomenological trends of both growth and decay of a microbial population in a given medium are easily reproducible with simple equations that allow gathering the experimental data (plate counts) related to different microbial species, in different mediums and even at different temperatures, in a single master plot. The guideline of the proposed approach is that microbes and surrounding medium form a system where they affect each other and that the so-called “growth curve” is just the phenomenological appearance of such interaction. The whole system (cells and medium) changes following a definite pathway described as the evolution of a “virtual” microbial population in planktonic conditions. The proposed equations come from the assumption of a duplication mechanism with a variable generation time for the growth and of an exponential-like decline with a linear increase of the rate for the decay. The intermediate phase between growth and decay is a time span during which growth and death counterbalance each other and age differences within the virtual cell population tend to level off. The proposed approach does not provide an *a priori* description of this phase but allows the fit of the whole evolution trend of a microbial culture whenever the experimental data are available. Deviations of such a trend concern microbes able to form spores, modify their metabolism, or express phenotypic heterogeneity, to counterbalance adverse medium conditions.

## 1. Introduction

Microbial populations undergo a number of changes that depend on the surrounding environment and the attained level of the population density itself. These changes affect the biochemical activity within each single cell (synthesis of nucleic acids, number of active ribosomes, synthesis of proteins, uptake of external resources, etc.) and the exchange of signals between neighbor cells (quorum sensing). The overall result of such changes is the phenomenological evolution (“growth curve”) of the population density, *N*, that goes through different “phases,” which correspond to observed changes of the specific rate $\dot{N}/N$ (where $\dot{N}$ stands for d*N*/d*t*, *t* being the time). $\dot{N}/N$ is null during the starting lag phase, positive in growth phase, again null during the intermediate stationary phase, and finally negative in the decay phase. $\dot{N}/N$ is both the cause and the effect of the biochemical activity of the cells. According to Neidhardt [1], “the macromolecular composition of bacteria is a monotonic function of the growth rate; the faster the growth rate, the larger the cells, the richer they are in ribosomes and t-RNA, and the greater their level of transcription and translation factors, including aminoacyl-tRNA synthetases.”

This observation is in line with the definition of the “balanced growth” (that approximately corresponds to so-called the exponential phase in the growth curve of batch cultures) as the regimen attained when “each cellular component increases by the same proportions in each interval of time.” [2, 3] This special physiological behavior is optimal for chemostat cultures that allow a more reliable comparison between various environmental conditions (different temperatures, pH, adverse substances, etc.) [4].

The same view accounts for the simultaneous presence of viable duplicating and not-duplicating cells within a given microbial culture and the possible conversion of the former toward the latter and vice versa. The neat balance between these subpopulations changes during the overall evolution of the culture because of the concomitant modification of the surrounding environment (available substrate, cell crowding, production of adverse catabolites, etc.), which can even induce sporulation of some bacterial species.

The adjustments of the cells actually are the result of coordinated processes at the macromolecular level that regulate the cytoplasm and membrane biochemical machinery through activation of some enzymes and repression of some others, following the so-called “passive control” regulation [5].

“Shifting cultures from a medium that affords a slow growth rate to one that leads to a higher rate results in a rapid acceleration of ribosome synthesis. The converse, going from fast to slow growth, imposes a long lag required for the synthesis of biosynthetic enzymes repressed in the rich medium. Both patterns could be partly understood in terms of the partitioning of the transcriptional and translational apparatus between synthesis of the repressible biosynthetic enzyme systems and making the protein synthetic system.” [3]

The above statements suggest that determination of the specific rate $\dot{N}/N$ is a major issue of the experimental approach to microbial physiology, as it directly reflects the biochemical changes within the single cell in response to the external environment, including the surrounding cells. The main trend of the cellular adjustments could therefore be quite the same for every duplicating microbial species, no matter the peculiarities of each single strain.

Since cells and environment seem to affect each other, the cells of a given inoculum poured into a new medium would “perceive” the new context and react consequently. In a favorable medium, they would adjust the uptake of nutrients to increase first the DNA and tRNA resources and then the number of ribosomes [1], someway planning the attainment of the balanced growth that is the most efficient behavior corresponding to the maximum specific growth rate. This state, however, would be transitory because of the concomitant changes of the medium (including the cell crowding) that would desynchronize the components of the biochemical machinery and reduce the specific growth rate, until the population density reaches the maximum sustainable level, *N*_max_.

In an adverse medium, the cells would search the best way to remain viable as long as possible, including the suppression of some surrounding sisters, before undergoing death or sporulation.

This means that some correlation should exist between duration of lag phase and maximum specific growth, as well as between duration of the stationary phase and specific decay rate.

The “perception” mechanism is largely unknown at present, but one may assume that it could likely imply a number of biochemical feedbacks. Nonetheless, the check of a correlation between “phases” of the growth trend seems more accessible at the phenomenological level through a growth model that can gather the behaviors of different microbial species in a single representation.

Previous papers [6, 7] allowed the achievement of this goal for lag phase and growth trend. The model [6] describes the virtual behavior of a batch planktonic culture of microbes in the presence of excess substrate, at constant temperature. Such a culture behaves like a closed system formed by two main components: the cells and the surrounding environment, the latter including the available substrate. The microbial population undergoes growth and death, while the surrounding medium undergoes changes (pH, accumulation of catabolites, etc.) which depend just on the growth and death processes, so as to comply with the evidence that cells and environment affect each other and that either component of the system plays a crucial role.

The model [6] assumes that growth occurs via duplication, namely, *N *=* N*_0_2^*t*/*τ*^ (*N*_0_ being the starting population density), and that the generation time, *τ*, is a property of the whole system, accounting for any endogenous change of the culture (including the death of some cells and changes of the environment during the growth span). It was found that the generation time may be described as *τ* = ((*a*/*t*) + *bt*), where the parameters *a* and *b* come from the best fit of experimental plate count data. A consequence of this choice is that the ratio (*t*/*τ*) is zero for *t* ⟶ 0 and tends to (1/*b*) for *t* ⟶ ∞. The starting *N*_0_ cells are supposed to have the same age and to stem *N*_0_ synchronous generation lines without experiencing any death process. After 1/*b* generation steps, this virtual population attains its maximum level *N*_max_ = *N*_0_2^1/*b*^. The maximum of the specific growth rate, $\dot{N}/N$, occurs at *t* = *t*^*∗*^ = (*a*/3*b*)^1/2^. One can finally describe the growth progress with a continuous trend, *N*=*N*_0_2^*ξ*(*t*)/*b*^, with 0 ≤ *ξ*(*t*) ≤ 1.

**Figure 1:**
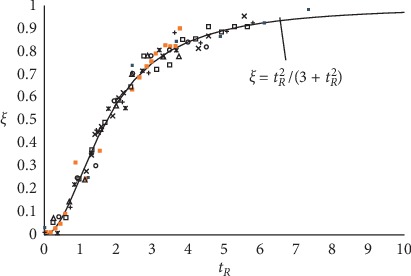
Growth trends of many microbial cultures in different environmental conditions gathered in a single plot of reduced variables. Reported from [6].

Since the parameters *a* and *b* come from the best-fit treatment of experimental plate counts, the model is substantially empirical. This allows the virtual behavior described above to match that of every real culture at any step of the growth. For this reason, one can gather the growth trends of all duplicating microbes in a single master plot of reduced variables, *ξ*(*t*_*R*_) and *t*_*R*_, where *t*_*R*_ = *t*/*t*^*∗*^ and *ξ*(*t*_*R*_)=(*t*_*R*_^2^/(3+*t*_*R*_^2^)) (Figure 1).

In a real system, the oldest cells die and newborn cells over-replace them until the cell density attains its maximum, *N*_max_. The proposed model does not explicitly account for cell death, the effect of which are actually concealed in the *τ*(*t*) function; nonetheless, age differences appear during the growth phase: at any time, *t*, the microbial population contains newborn and aged cells, the oldest ones being those of the starting inoculum, *N*_0_.

The advantage offered by the model is that, because of the algebraic relationships between *t*^*∗*^, *N*^*∗*^, and *N*_max_, an explicit correlation can be found between the duration of the lag phase and the maximum specific growth rate [7]: the larger the maximum specific growth rate, the shorter the lag phase.

The proposed model therefore seems suitable to describe the lag phase and the overall increase of the population density up to *N*_max_ but cannot describe what happens later, when growth and death counterbalance each other and the overall average number of viable cells remains constant (actually, fluctuations do occur [8, 9]) for some time span and eventually starts to decline.

The present paper completes the description including the decay phase which again requires a model to describe the behavior of many microbial species gathering all them in the same master plot representation. Finally, coupling the expressions for the growth and the decay phases, an equation is proposed for the whole growth-steady-decay trend that satisfactorily fits the experimental data reported in the literature.

## 2. Materials and Methods

All the data used in the present work come from literature. They concern cultures of various duplicating microbes in the presence of excess substrate, at constant temperature, in broth or broth-like medium. Most of them are reported in the COMBASE archive, where the experimental details are fully described. The same holds for the data from quoted papers.

For the present work, these data were transferred in EXCEL worksheets with the simplest format, namely, a two-column array for *t* and *N*, respectively, for each considered microorganism. This format allowed the direct transfer of the data to the TABLECURVE (Jandel Sci.) program for the best fit treatment. In most cases, a preliminary fit of the data with any sp-line was of help to find tentative values for the fitting parameters of the model, like *t*^*∗*^, namely, the time at which $\dot{N}/N$ goes through a maximum during the growth phase, or $\dot{N}$ goes through a minimum during the decay phase (see below). The suggested procedure to transfer experimental decay trends into a master plot in reduced variables is reported in Section 3.

## 3. Results and Discussion

### 3.1. Decline of the Cell Population

One should notice that the population density goes through equal values along the rising and declining path although the system is clearly different in the two states. When the population is rising, the environment sustains the growth, while when the population is declining the environment favors the cell death. However, when a small amount of the decaying microbial culture is poured into a fresh environment, the cell growth would reignite after a lag phase. Once again, cells and environment seem to affect each other. Description of the decline behavior should therefore account for both characters of the system, just as for the preceding growth trend.

A virtual behavior can again apply, accounting for the main experimental evidence that will dictate the values of the parameters of the model. A simple virtual behavior could imply the following:

1. Age differences level off during the stationary phase preceding the onset of the decline. Such a behavior would look like that during the lag-phase when the inoculum cells adjust to the new surrounding environment.
2. The concomitant changes of the medium (exhaustion of the substrate, pH change, accumulation of catabolites, etc.) finally do not allow any further duplication and the death rate governs the number of viable cells.

Experimental evidence shows that the decay progress occurs with an increasing pace that may suggest an exponential-like trend. In the present case, the decay has to start from *N*_max_ = *N*_0_2^1/*b*^ and end at *N* = 0. It therefore seems reasonable to describe the decay trend with a continuous function *N*_*d*_(*t*) that accounts for the number of dead cells:

(1) $$\begin{array}{c} N_{d}\left(t\right)=N_{\max}\left(1-\exp\left[\varphi \left(t\right)\right]\right), \end{array}$$

with the contour conditions, *φ*(*t *⟶* *0) = 0 and *φ*(*t *⟶* *∞) = −∞.

A simple choice for *φ*(*t*) is

(2) $$\begin{array}{c} \varphi \left(t\right)=-\frac{t^{\delta }}{d}, \end{array}$$

where the parameters *d* and *δ* reflect the combined cell/environment effect that makes the decay rate to increase, both quantities being positive. The validation tests with experimental data found in the literature (see below) suggest *δ* = 2 to be the best choice. With respect to the simple exponential decay, exp(−*kt*), putting *δ* = 2 is tantamount to saying that *k* increases with a straight-line trend. The number of surviving cells therefore is

(3) $$\begin{array}{c} N_{\text{surv}}\left(t\right)=N=N_{\max}\exp\left[\varphi \left(t\right)\right]=N_{\max}\exp\left[-\frac{t^{2}}{d}\right]. \end{array}$$

**Figure 2:**
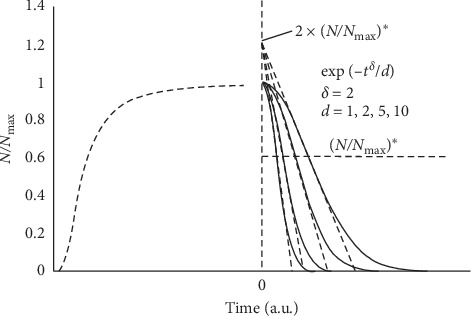
Decay trend of a planktonic microbial culture. The different trends correspond to various values of the parameter *d* (1, 2, 5, and 10 time-units^2^) for *δ* = 2. The dashed curve on the left reflects a hypothetical growth trend leading to *N*_max_.

Figure 2 reports the corresponding decay profiles for various *d* values.

A straightforward algebra lets one to realize that the decay rate, $\dot{N}$, goes through a minimum for

(4) $$\begin{array}{c} t=t^{*}={\left(\frac{d}{2}\right)}^{1/2}. \end{array}$$

The corresponding value of *N* does not depend on the value of *d* (Figure 2):

(5) $$\begin{array}{c} N^{*}=N_{\max}\exp\left(-\frac{1}{2}\right). \end{array}$$

The tangent straight lines through *t*^*∗*^ have the same intercept at *t* = 0 (Figure 2):

(6) $$\begin{array}{c} y\left(0\right)=2\;\exp\left(-\frac{1}{2}\right)=2\times {\left(\frac{N}{N_{\max}}\right)}^{*}. \end{array}$$

It is worth noting that the corresponding specific decay rate, $-\left(\dot{N}/N\right)=-\left(\text{d}/\text{d}t\right)\log\left(N\right)$, has no maximum, i.e., the corresponding trend does not show any inflexion point, which makes a remarkable difference with respect to other decline models that allow the occurrence of either upward or downward change of the relevant curvature [10–12].

**Figure 3:**
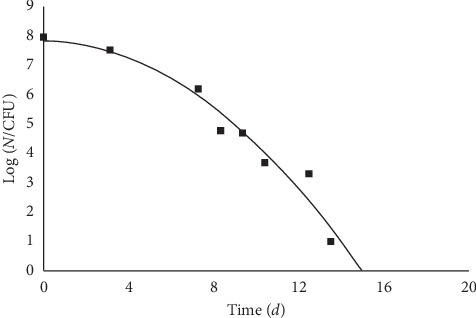
*Escherichia coli* at 10°C, pH 4 in the presence of 0.7% oregano essential oil. Plate count data after Skandamis and Nychas, as reported in [13].

Equation (3) is a suitable tool to fit experimental data related to the effects of adverse conditions (pH, bactericidal substances, drugs, rise of temperature, etc.) that trigger and/or accelerate the cell death. The literature abounds of data related to the decay of microbial populations under the effects of some thermal or chemical treatment. An example may be the decline of an *Escherichia coli* culture in the presence of oregano oil [13].  Figure 3 reports the relevant best fit according to equation (3).

Equations (4) and (5) allow an alternative form of equation (3):

(7) $$\begin{array}{c} \frac{N}{N^{*}}=\exp\left[-\frac{1}{d}t^{2}+0.5\right]. \end{array}$$

Introducing the reduced variables, *t*_*R*_ = *t*/*t*^*∗*^, and Δ = log_*e*_(*N*/*N*^*∗*^), one obtains

(8) $$\begin{array}{c} \Delta =\frac{1}{2}\left(1-\;t_{R}^{2}\right). \end{array}$$

The relevant straight line tangent at *t*_*R*_ = 1 is

(9) $$\begin{array}{c} y\left(t_{R}\right)=1-t_{R}. \end{array}$$

**Figure 4:**
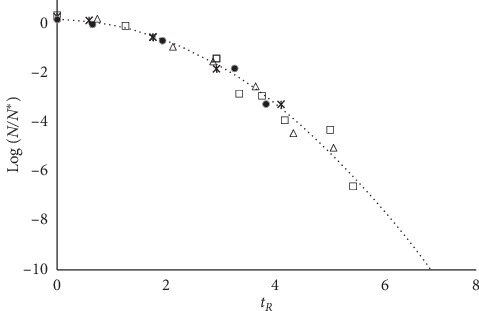
Master plot gathering the experimental data for *E. coli* (open squares), *Listeria* (stars), *Salmonella* (open triangles), and *Staphylococcus aureus* (full circles) [14].

Equation (8) allows one to gather the decay trends for any microbial population, no matter the value of *d*, in a single master plot Δ-*vs*-*t*_*R*_.  Figure 4 reports the decay data for some microbial strains from the COMBASE data bank.

The suggested procedure to transfer experimental data relevant to decay trends into the master plot in reduced variables is as follows:

1. Fit the decay data with any sp-line and single out the *t* value at which log_*e*_(*N*)= log_*e*_(*N*_max_) − 0.5; this is a tentative value for *t*^*∗*^ that allows a tentative estimation of the parameter *d* through equation (4)
2. Fit again the data with equation (3) to adjust the values of *N*_max_ and *d*
3. Calculate log_*e*_(*N*^*∗*^)=(log_*e*_(*N*_max_) − 0.5) and scale the data accordingly (and adjusting for the use of log_10_ scale)

**Figure 5:**
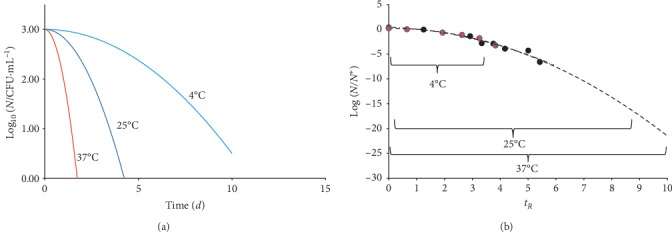
Decay trends of *E. coli* in lager beer at different temperatures. (a) The curves correspond to equation (3) and closely describe the trend of the experimental data reported in [15]. (b) The same data gathered in a single master plot of reduced variables, according to equation (8), matched with those relevant to *E. coli* (full circles) reported in Figure 3.

Figure 5 reports a further example.

One may therefore guess that the microbial cultures gatherable in the above plot would undergo the population decay following the same pathway.

**Figure 6:**
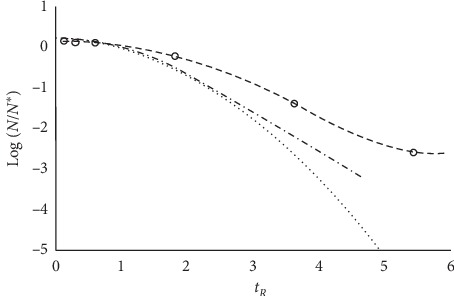
Deviation from the decay model (dotted curve) observed for *Clostridium* in broth at 85°C (open circles) [16] and *Pseudomonas aeruginosa* at 37°C (point-dotted curve) [17].

However, some microbial species, once facing adverse conditions that make the population density to decrease, adopt counter measures, like, for instance, formation of spores, as in the case of *Clostridium* [16], or modification of the cellular metabolism (from aerobic to anaerobic or vice versa, etc.) or express a phenotypic heterogeneity, as in the case of *Pseudomonas aeruginosa* [17]. The result is a decay rate smaller than expected and the appearance of an inflection point in the log (*N*)-*vs*-*t* decay trend (Figure 6).

Because of such “exceptions,” which are of some relevance for the importance (human health and food and soil contamination) of the microbial strains involved, one may not give to the decay master plot in Figure 4 the same general character as to the growth master plot (Figure 1). Nonetheless, deviations from the decay master plot (Figure 6) could be the phenomenological evidence of some biochemical peculiarity of the corresponding cell-environment system and alert researchers to look after it.

### 3.2. Growth and Decay

As mentioned above, the present model does not imply a description of the gap between rise and decline trend save for the assumption that during this intermediate phase, the cell population undergoes an age leveling. Figure 2 reports a tentative growth trend preceding the decay, although with an explicit lack of continuity. In order to match growth and decay trend, one has to rewrite the relevant equations and express both trends with reference to their common value, namely, *N*_max_, and define a single time scale with the zero point at the onset of the growth. It is easy to adjust the equation for the growth trend that becomes

(10) $$\begin{array}{c} N=N_{\max}\times 2^{-\left(\left(\gamma /b\right)/\gamma +t^{2}\right)}, \end{array}$$

where *γ* = *a*/*b*.

Some problems instead concern the decay trend since the model assumes no cell death before the attainment of *N*_max_. However, the desired effect comes from selecting large *d* values (i.e., negligible decay rate in the time range of prevailing growth) so that one may use the following expression:

(11) $$\begin{array}{c} N=\;N_{\max}\times 2^{-\left(\left(\gamma /b\right)/\gamma +t^{2}\right)}\;\times \;\;\exp\left[-\frac{{\left(t\right)}^{\delta }}{d}\right], \end{array}$$

that encompasses the whole evolution of the microbial culture.

**Figure 7:**
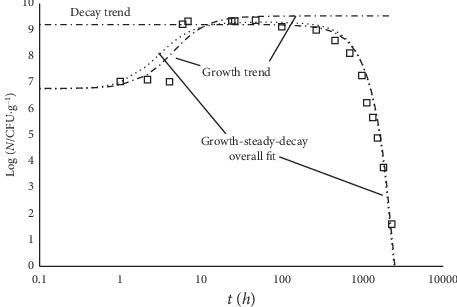
Evolution of an *E. coli* culture in feta cheese at 15°C and starting pH 6.75 [18]. The fitting curve (growth-steady-decay overall fit; point line) corresponds to equation (11). Log_10_ (*N*_max_/CFU·g^−1^) = 9.3; *γ* = 8.7 *h*^2^, *d* = 2.75 10^5^*h*^2^, *b* = 0.11, and *δ* = 2. The figure also shows the growth and the decay contributions. Because of the large time span covered by the data, the variable *t* is in log_10_ scale.

While the literature abounds of experimental data about just the decay trend, there are scanty case studies that report the whole evolution of a microbial culture, which would indeed be of great interest.  Figure 7 reports a case study of an *E. coli* culture [18].

**Figure 8:**
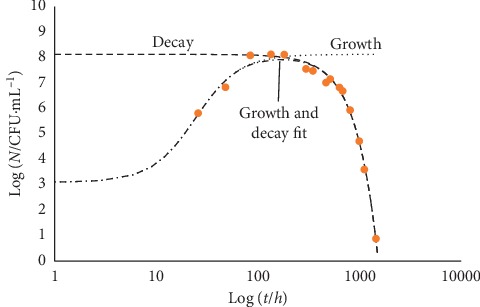
Growth and decay trend of *Salmonella* in broth. The experimental data come from the COMBASE data bank (Com Base ID: B10_1). The fitting curves correspond to equation (11). Log_10_ (*N*_max_/CFU·mL^−1^) = 8.12, *γ* = 589 *h*^2^, *b* = 0.06, *d* = 1.25 10^5^*h*^2^, and *δ* = 2. Because of the large time span covered by the data, the variable *t* is in log_10_ scale.

Looking at Figure 7, one can realize that a practically steady level of the microbial population encompasses a wide intermediate gap (about 1000 hours) between rise and decline trends: it possibly reflects the combined effect of cell duplication in the presence of substrate shortage, excess of cell crowding, and chemical and/or physical changes of the medium. Extra data could allow one to single out the major responsible for the decay onset.  Figure 8 reports the case of *Salmonella* in broth.

The comparison between Figures 7 and 8 allows recognition that the decay trend encompasses practically the same time range for either microbial culture, while the intermediate phase between growth and decay of *Salmonella* is much shorter than in the case *E. coli* (Figure 7) and its growth trend is much slower.

**Figure 9:**
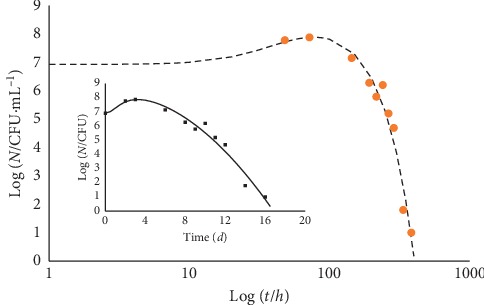
*Escherichia coli* at pH 4.5 in the presence of oregano oil. Plate count data after Skandamis and Nychas [13]. For the sake of comparison with the previous figures, the variable *t* is in log_10_ scale. The insert reports the same data in the time units used in [13].

Figure 9 shows the case of *Escherichia coli* in the presence of oregano essential oil at pH 4.5 [13].

**Figure 10:**
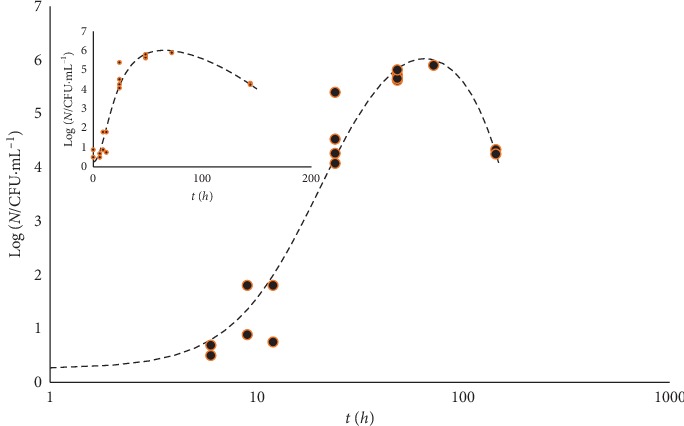
Growth and decay trend of *Listeria monocytogenes*. The experimental data come from the COMBASE data bank (Com Base ID: Lm_Nat_25C_T3). The fitting curves correspond to equation (11). Log_10_ (*N*_max_/CFU·mL^−1^) = 7.25, *γ* = 429 *h*^2^, *b* = 0.14, and *d* = 7.3 10^3^*h*^2^ and *δ* = 2. The insert reports the same data in the time units used in the source.

Figure 10 reports the growth and decay trend of *Listeria monocytogenes*/*innocua* in milk shake.

In the latter two cases, the intermediate phase between growth and decay appears like a maximum of the fitting curve, no matter what scale is used for the variable *t*.

**Figure 11:**
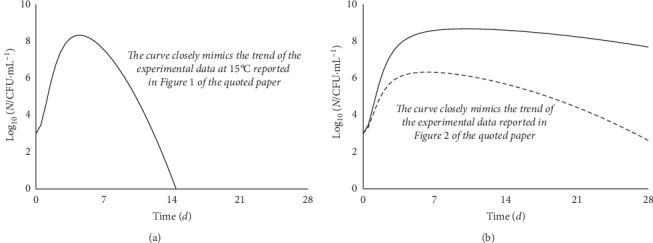
Growth and decay trend of *E. coli* in low-fat milk, data after [19]. (a) Pasteurized milk kept at 22°; (b) pasteurized (full line) and unpasteurized (dotted line) milk kept at 15°C. The curves correspond to equation (11) and closely reflect the trend of the experimental data (not reliably transferable from the figures of [19]).

Figure 11 shows the trends reported for *E. coli* in low-fat milk [19].

The last case study again shows a maximum trend between prevailing-growth and prevailing-decline trends, the broadness of which seems to be strongly affected by the temperature, although this physical parameter does not modify the overall behavior. Consequently, one may guess that the temperature simply affects the rate, but not the kind, of the underlying biochemical processes.

Similar situations occur in the presence of adverse substances added to the microbial cultures. In these cases, the extent of the maximum seems related to the concentration of the antimicrobial compound(s) [20].

## 4. Conclusions

A semiempirical model allows description of growth and decay trends of a microbial culture accounting for the accompanying modification of the surrounding medium, referred as to a co-partner in the evolution of the whole system.

For both growth and decay phases, the model allows a representation that gathers all the microbial species in a single master plot of reduced variables whenever the considered system is left to spontaneously evolve, starting from given initial conditions, without external perturbations.

As for the decay trend, the model does not account for adjustments of the microbial physiology, like sporulation, phenotypic heterogeneity, and aerobic/anaerobic tolerance, adopted by the microbial population to match the adverse medium conditions and reduce the decline rate. In such cases, deviation from the predicted behavior is a simple phenomenological evidence of the underlying change of biochemical and/or genomic activity.

A simple modification of the model equations for the growth and decay phase allows the definition of a new equation that describes the whole evolution of the microbial culture, including the intermediate pseudosteady state that would mainly imply an age leveling of the microbial population.

## Data Availability

The data used to support the findings of this study are included within the article.
